# MMPs in learning and memory and neuropsychiatric disorders

**DOI:** 10.1007/s00018-019-03180-8

**Published:** 2019-06-06

**Authors:** Anna Beroun, Shiladitya Mitra, Piotr Michaluk, Barbara Pijet, Marzena Stefaniuk, Leszek Kaczmarek

**Affiliations:** BRAINCITY, Nencki Institute, Pasteura 3, 02-093 Warsaw, Poland

**Keywords:** Behavioral training, Schizophrenia, Autism, Epilepsy, Addiction

## Abstract

Matrix metalloproteinases (MMPs) are a group of over twenty proteases, operating chiefly extracellularly to cleave components of the extracellular matrix, cell adhesion molecules as well as cytokines and growth factors. By virtue of their expression and activity patterns in animal models and clinical investigations, as well as functional studies with gene knockouts and enzyme inhibitors, MMPs have been demonstrated to play a paramount role in many physiological and pathological processes in the brain. In particular, they have been shown to influence learning and memory processes, as well as major neuropsychiatric disorders such as schizophrenia, various kinds of addiction, epilepsy, fragile X syndrome, and depression. A possible link connecting all those conditions is either physiological or aberrant synaptic plasticity where some MMPs, e.g., MMP-9, have been demonstrated to contribute to the structural and functional reorganization of excitatory synapses that are located on dendritic spines. Another common theme linking the aforementioned pathological conditions is neuroinflammation and MMPs have also been shown to be important mediators of immune responses.

## Introduction to MMPs in the brain

Extracellular matrix (ECM) in the brain has emerged as an important reservoir of signaling molecules, which can influence synaptic plasticity, synaptogenesis, neurite outgrowth and other processes occurring in central nervous system. Many suggest now that “tetrapartite synapse” is a functional unit of the brain, where ECM plays equally important roles to pre-, post-synaptic terminals and glial cells’ invaginations [1, 2]. Matrix metalloproteinases (MMPs) are among the major modulators of ECM, providing precise proteolysis of its components, in addition to performing limited cleavage of cell adhesion molecules (CAMs), neurotrophins and cytokines [3, 4]. MMPs make up a family of over 20 proteins in humans and rodents (predominantly secreted, but six are membrane-bound), each encoded by a different gene. MMPs are a part of bigger, metzincin group of proteases, which all have Zn^2+^ and the conserved methionine residue in their active site [3, 5].

Based on their domain structure, MMPs are subdivided into eight groups, but all are composed of (starting from N-terminus): a signal peptide, propeptide, catalytic domain and in most cases hinge region and hemopexin domain. The signal peptide is removed after the protein enters the endoplasmic reticulum. MMPs are expressed as inactive zymogens, where the propeptide has conserved cysteine residue binding Zn^2+^ in the active center of the enzyme. Activation occurs when the interaction between cysteine and Zn^2+^ is disrupted, which can be achieved by cleaving the propeptide off (by other proteases), as well as by other means, such as S-nitrosylation, detergents or sulphates [5, 6]. The catalytic domain of all MMPs has a highly conserved Zn^2+^ binding motif—HExGHxxGxxH (where x is any amino acid) and a distinct β-turn (usually ALMYP). That is why all MMPs have distinguishable, but often overlapping, substrate specificities. Moreover, the catalytic domain of MMPs requires binding of Ca^2+^ ions which are required for the stabilization of its active state [7]. Gelatinases (MMP-2 and -9) have additional fibronectin type II inserts, which are required for collagen and elastin binding and cleavage.

Most of MMPs (except of MMP-7, MMP-26 and MMP-23) have hinge region and hemopexin domain, which is responsible for interaction with substrates, dimerization and binding of one of four endogenous tissue inhibitors of metalloproteinases (TIMPs). MMP-23 instead of hemopexin domain has unique cysteine-rich, proline-rich and IL-1 type II receptor-like domains. MMP-14, MMP-15, MMP-16, and MMP-24 have also a transmembrane domain which anchors them to the cell membrane whereas MMP-17, and MMP-25 have on their C-terminus a glycophosphatidyl inositol membrane anchoring signal [5].

Since excessive activity of MMPs might be detrimental to the tissue (as it happens under some pathological conditions), the enzyme gene expression, mRNA maturation, distribution and survival, as well as protein release and activation are all strictly regulated. This can be best described in the example of one of the most studied MMP, MMP-9 [8]. MMP-9 transcription is activity regulated by inducible transcription factors, such as AP-1 [9, 10], then MMP-9 mRNA transport, survival and translation are also activity regulated, as it has been demonstrated for neurons [11–13]. MMP-9 is present within cells in vesicles distributed along microtubules and microfilaments and is secreted in a Golgi-dependent pathway in 160–200 nm vesicles [14–16]. Notably, MMP-9 is often found in the same secretory vesicles as its inhibitor, TIMP-1 and although secreted upon stimulation, it is thought to act very focally and can be concentrated at the cell membrane by binding to cell adhesion molecules such as the hyaluronian receptor CD44 [17, 18] or integrins [19].

The activation of proMMP-9 occurs extracellularly after its release and is controlled by a cascade of steps involving other proteases, inhibitors and receptors. For example, serine proteases: tissue plasminogen activator (tPA) or urokinase plasminogen activator (uPA), which can be docked to the membrane by its receptor, uPAR, activate the conversion of plasminogen to plasmin, which in turn can activate proMMP-9 [20]. Additionally, some MMPs such as MMP-11 or MMP-28 and all membrane-type MMPs (MT-MMPs) have also a furin-like recognition motif in their propeptide and can be thus activated inside the cell before secretion or exposure to cell surface, and activate other secreted MMPs [21]. Once activated, MMP-9 can be inhibited, particularly by TIMP-1, but also by other TIMPs [22]. This is because, similarly to redundancy in MMPs substrate specificity, there is a significant overlap in the affinity of TIMPs to different MMPs [8].

TIMP-1 can inhibit MMPs directly by binding through its N-terminal domain to the catalytic site of the enzyme [23]. Interestingly, inhibition of MMP-9 can be also achieved by binding TIMP-1 C-terminus to the hemopexin domain of MMP-9, thus blocking its interaction with substrate or membrane docking proteins [24]. MMP-9 and TIMP-1 share the same release kinetics [25] and it has been shown that those two proteins can be co-released even from a single vesicle [14, 15, 26]. Moreover, it has been proposed by Ogata and co-workers [27] that TIMP-1 bound to MMP-9 via the hemopexin domain is also important during activation of the gelatinase by other MMPs, like MMP-3. Finally, activated MMP-9 binds through its hemopexin domain to low-density lipoprotein receptor-related protein, which is an endocytic receptor for this and many other proteins, and after endocytosis leads to the destruction of MMP-9 [28].

All the regulatory mechanisms, such as secretion, interaction with membrane receptors, internalization and TIMP-dependent inhibition, are to limit spatially and temporarily MMP’s activity outside the cell and prevent excessive, potentially pathological activity. An interesting example of physiological, tight control by TIMP-1 over MMP-9 has been recently published by Magnowska et al. [29], where both proteolytic activity of MMP-9 and its following inhibition by TIMP-1 were discovered to be necessary for synaptic plasticity (see below). Interestingly, activation of MMP-9 in the brain tissue can be achieved by the degradation of TIMP-1 by cathepsin B, a lysosomal enzyme, which upon stimulation can be released to extracellular space and activate MMP-9 [30, 31]. Another example of MMP activating cascade is that of MMP-2, as it requires the formation of tertiary complex with TIMP-2 and MT1-MMP [32]. TIMP-2 forms a link between dimerized MT1-MMP and the hemopexin domain of pro-MMP-2. This leads to cleavage of MMP-2 propeptide by MT1-MMP and thus its activation.

As already mentioned, MMPs are inhibited by four endogenous inhibitors, TIMP-1 to TIMP-4. They are all soluble and share a common structure, but their role is very diverse. For example, TIMP-1 can act independently to its inhibitory activity as a growth factor suppressor [33] or a cytokine [34]. The cytokine effect of TIMP-1, which results in inhibition of neurite outgrowth and increase in growth cone volume, is mediated through its internalization by LRP-1 [35, 36].

## Expression in the brain

Among the MMPs, many have been shown to be expressed in the brain, namely: MMP-1, MMP-2, MMP-3, MMP-7, MMP-8, MMP-9, MMP-10, MMP-11, MMP-12, MMP-13, MMP-14, MMP-15, MMP-16, MMP-17, MMP-24 and MMP-28. Notably, a number of those are expressed in the brain only under very particular physiological and/or pathological circumstances.

MMP-1 (collagenase 1) expression in the brain has often remained undetected [37, 38]. However, recent studies have shown MMP-1 immunoreactivity in the olfactory lobe, entorhinal cortex, pontine nuclei, and periaqueductal gray, but not in the hippocampus [39]. Moreover, MMP-1 protein level was increased at 4 h following systemic kainate administration [39] or after ischemic stroke [40], and was shown to be present in the brains of Alzheimer’s disease patients [41]. MMP-1 seems to be mostly expressed by glia [42, 43], but there are also reports indicating its presence in neurons [39]. MMP-1 was also shown to enhance proliferation and increased differentiation towards neurons of hippocampal neural progenitor cells [44]. Moreover, MMP-1 overexpression in transgenic mice under GFAP gene promoter enhances dendritic complexity and causes deficits in learning and memory [42]. Literature suggests that MMP-1 in the brain acts mainly through activating protease activated receptor 1 (PAR1) [44], and thus increasing cytoplasmic Ca^2+^ concentration [42], as it was proven also in platelets [45]. Interestingly, PAR1 was shown to be cleaved and activated by another collagenase which is also expressed in the brain, MMP-13, however, this relationship was studied in cardiac fibroblasts and cardiomyocytes [45].

MMP-2 (gelatinase A) is one of the two gelatinases most extensively studied in the brain. It is constitutively expressed mainly by astrocytes [46, 47], however, many studies find it to be localized also in some cortical, and cerebellar neurons, as well as in the hippocampus and nucleus accumbens (NAc) [46, 48–51]. MMP-2, like most of the MMPs in the brain, is expressed at much higher level during early postnatal development than during adulthood, reaching peak around day 4 after birth [48, 52]. MMP-2 expression can be induced under various pathological conditions (see below). In particular, reactive astrocytes show increased expression of MMP-2 [53]. MMP-2 in astrocytes is localized at the leading edge of processes, where it regulates actin cytoskeleton and thus affects cell migration [54].

MMP-3 (stromelysin 1) is, as many other MMPs, expressed during development in the central nervous system. Around embryonic day 15, it is expressed in most brain areas in rat, and in mouse it is found in neurons of the ventricular zone. In late prenatal period, MMP-3 is also expressed in mature oligodendrocytes [55]. Until postnatal day 10, MMP-3 is expressed by most cells of the cerebellar cortex, striatum and hippocampus and can be found in both neuronal and glial cells [55–57]. MMP-3 is also expressed during postnatal brain development in cerebellum, specifically in Purkinje cell somata and dendrites as well as in some granule cells [51, 58]. In the adult brain, MMP-3 expression level is either very low or negligible [37, 59], but some studies report its expression in the hippocampus, especially during learning paradigms [60, 61]. A recent study of Wiera et al. [57] shows that in the stratum radiatum of hippocampus, MMP-3 seems to be mainly expressed by astrocytes in a sparse punctate manner. MMP-3 has a very broad range of substrates in the brain. It was shown, for example, to cleave almost all components of perineuronal nets [53]. MMP-3 is upregulated in many brain pathologies, disrupting blood–brain barrier, causing neuroinflammation and apoptosis. In such conditions, it is expressed by injured neurons, oligodendrocytes, astrocytes and reactive microglia or invading macrophages [62, 63]. Induction of MMP-3 expression depends on binding of NFκB to its gene promoter upon the release of inflammatory mediators, e.g., interleukin-1β caused by ischemia, traumatic brain injury (TBI) or infections [64]. In addition, MMP-3 acts itself as a signaling molecule and creates positive feedback for inflammatory response, as it causes activation of microglia and synthesis of cytokines, e.g., tumor necrosis factor-α, interleukin-1β, or interleukin-6 [65]. However MMP-3 was also shown to have beneficial effects during pathological states as it can cleave Fas ligand [66], can promote synaptogenesis [67] and is involved in remyelination [68].

MMP-7 (matrilysin) expression during development, as well as in the normal adult brain, is often described as negligible [59, 69–71]. Most reports that detect MMP-7 in normal brain conditions point to glia (most often microglia) as MMP-7 expressing cells [69, 72, 73]; although the issue of MMP-7 source in the brain has often not been addressed in various studies. Interestingly, Le and Friedman [74] reported a high level of MMP-7 in hippocampal neurons, which dropped after seizures induced with kainic acid. MMP-7 is, though, strongly upregulated during such pathological conditions in the brain as multiple sclerosis [73], experimental autoimmune encephalomyelitis [75], HIV dementia [76] or brain tumors [71]. Among the substrates of MMP-7 in the brain, the most prominent are pro-nerve growth factor (pro-NGF) [74, 77], SNAP-25 [78], the NR1 subunit of NMDA receptor [79] or myelin-associated glycoprotein [80].

MMP-9 (gelatinase B) is the most studied MMP in the brain, with its expression in naïve brain kept on a very low level. It is found in hippocampus, cerebellum and cerebral cortex [46, 47, 51, 52] and is predominantly expressed by neurons, but also by glia [15, 47]. MMP-9 expression is tightly regulated on different levels (see above) and it peaks during early development [48, 51, 52]. Even though MMP-9 levels in the adult brain are low, they are upregulated during neuronal increased activity/plasticity but also, as so many other MMPs in pathological situations (see below and for recent reviews: [4, 81]). Most interestingly, MMP-9 activity, as well as its mRNA are present in the vicinity of synapses [11, 82–84].

MMP-14 (MT1-MMP) is most often studied in the brain in the context of gliomas, where it is highly expressed along other MMPs. Under pathological and inflammatory conditions, it has been found expressed by microglia [85]. MT1-MMP is, however, expressed on a low level in the normal adult brain [37]. MT1-MMP in the hippocampus is predominantly localized in neuronal cell layer [86] and expression of MT1-MMP is elevated upon BDNF treatment in mixed neuronal cultures [87]. In retina, it is expressed in retinal ganglion cells as well as in Müller glia [88]. Interestingly, MT1-MMP expression increases in female hippocampus upon the presentation of novel odor [89].

MMP-24 (MT5-MMP) is expressed at relatively high levels in the adult brain, particularly in the cerebellum, but also hippocampus and olfactory bulb [90, 91]. It can be found predominantly in neurons and its expression is increased during development regulating axonal growth and dendritic tree formation of Purkinje cells [91, 92].

## Synaptic plasticity, learning and memory: MMPs expression and function

Brain plasticity refers to the capacity for structural and functional reorganization of the neural networks in response to external challenges. Beside learning and memory, which allow for adaptation to the changing environment, neuroplasticity is necessary to recover after disorders and injuries. Brain plasticity is manifested at many levels in the nervous system, ranging from molecular events, such as changes in gene expression, protein availability and function, to cellular physiology, to behavior. At the cellular level, the plasticity is supported by dynamic modifications in neural connectivity and excitability that are driven by molecular changes in synapses, entire neurons and glial cells.

Synapses are particularly prone to dynamic alterations, and thus believed to play a major role in the plasticity. Aberrant synaptic plasticity leads to many pathological conditions, e.g., epileptogenesis, drug addiction, autism spectrum disorders, schizophrenia or depression. Compensatory brain plasticity may reduce the detrimental effects of such pathologies as multiple sclerosis, Parkinson’s disease, cognitive deterioration or Alzheimer’s disease. Induction and maintenance of activity-dependent synaptic plasticity adaptations require a temporal and spatial control of a complex sequence of events that result in the modification of pre- and postsynaptic content, as well as remodeling of the entire tetra-synapse morphology.

Excitatory synapses that are particularly plastic, are predominantly located on small dendritic protrusions called spines, whose morphology can be taken as a proxy for the synaptic efficacy [93]. Small dendritic spines often harbor silent synapses, equipped with a limited number of NMDA receptors for glutamate and virtually missing AMPA receptors (AMPAR) for this excitatory neurotransmitter. Increased synaptic efficacy that may produce neuronal depolarization associates with the accumulation of AMPAR (and thus enhanced glutamate responsiveness) that in turn correlates with increased spine volume and enlargement of the dendritic spine head, producing spines with a mushroom shape.

MMPs have repeatedly been implicated in synaptic plasticity thanks to their capacity to act in a tightly controlled, short temporal window, targeting extracellular matrix, cell adhesion molecules, synaptic receptors, neurotrophins and other proteins implicated in synaptic efficacy and remodeling. Studies with broad spectrum MMPs inhibitors have demonstrated impairments in various short- and long-term plasticity models including paired pulse and theta burst facilitation, maintenance of long-term potentiation (LTP), induction and magnitude of long-term depression (LTD) in the hippocampus [94–96], as well as ocular dominance plasticity in the visual cortex and barrel cortex plasticity, resulting from sensory deprivation [97–99].

Recent studies have shed light on the role of MMPs in synaptic plasticity. In this regard, the best studied involvement of metalloproteinase function in synaptic plasticity processes concerns the activity of MMP-9. This issue has been covered by extensive reviews [4, 100–103], so it will be only briefly presented here.

MMP-9 is released from the postsynaptic compartment of excitatory synapses in an activity-dependent manner [11, 82]. Upon activation, MMP-9 cleaves (among others) such synaptic cell-adhesion molecules as: β-dystroglycan [104], neuroligin-1 [105], nectin-3 [106], intercellular adhesion molecule-5 (ICAM-5) [107, 108], synaptic cell adhesion molecule-2 and collapsin response mediator protein-2 [109], affecting NMDA receptors mobility and function at the synapse [110, 111]. Chemical LTP experiments on dissociated and organotypic hippocampal cultures revealed MMP-9-dependent spine enlargement, accumulation of AMPA receptors in the synapse and increase in the spike count and bursts frequency [84, 112–115]. Transient function of MMP-9 is a requirement for maintenance of the late phase, NMDA-dependent LTP at various pathways in the hippocampus [116–119], prefrontal cortex (PFC) [120], as well as basal and central nuclei of the amygdalar complex [121]. Furthermore, Dziembowska et al. [11] reported increases in MMP-9 mRNA levels in the hippocampal dentate gyrus of rats with LTP evoked therein by the perforant path stimulation.

The increase of limited MMP-9 activity at the synapse has a profound impact on synaptic morphology. It was reported that activation of this metalloproteinase during LTP induction leads to the enlargement of dendritic spines [118]. This, actually, is a two-step process that begins with the MMP-9-mediated elongation of dendritic spines into apparently more immature, filopodium-like shape [122–124]. Then, only upon inhibition of the proteolytic activity, which in neurons might be mediated via specific inhibitor TIMP-1 [46, 125], dendritic spines assume larger, mushroom-like shape [29].

As far as learning and memory are concerned, MMP-9 activity was reported to be increased after Morris water maze, head-shake response, passive avoidance and appetitive learning [60, 61, 116, 126]. Importantly, in addition to the functional role of MMP-9 in activity-dependent synaptic remodeling, blocking the enzyme has dramatic consequences on learning processes in vivo. Using either pharmacological inhibition or genetic ablation, it was demonstrated that MMP-9 activity was necessary for hippocampus-dependent learning, as shown in Morris water maze, contextual fear conditioning and reconsolidation of fear memory [60, 117, 127, 128], as well as amygdala-dependent positive reinforcement conditioning in sucrose preference task [126]. On the other hand, fear conditioning to an auditory cue, as well as avoidance learning of an exposure to an air-puff or a bitter taste in the Intellicages, remained undisturbed in MMP-9 KO mice [117, 126]. Of note, LTP to lateral nucleus of the amygdala is believed to support the aversive learning, such as aforementioned fear conditioning to an auditory cue [121]. Therefore, lack of MMP-9 requirement in the aversive learning might be explained by the fact that the enzyme is not obligatory for the lateral amygdala synaptic plasticity and LTP.

Interestingly, MMP-9 has also been considered as a player in human cognition. In the study of Bach et al. [129], blocking the activity of MMP-9 by administration of its inhibitor doxycycline 3.5 h prior to the fear conditioning procedure, reduced the fear response in human subjects measured 1 week after the test. This might suggest that lower MMP-9 activity during fear acquisition phase attenuated the formation of the fear memory. Moreover, reduced mRNA and protein levels of MMP-2, MMP-9 and TIMP-2 were observed in patients diagnosed with depression and displaying lower performance in cognitive tasks, as compared to healthy subjects [130]. Furthermore, within the healthy control group, there was a positive correlation between MMP-2 (protein level), MMP-9 and TIMP-2 (mRNA levels) and performance in cognitive tasks: visuospatial performance, working memory tasks, auditory-verbal memory, the effectiveness of learning processes and verbal fluency [130]. Similar positive correlation between MMP-9 serum level and decision-making abilities was observed in bulimia nervosa patients [131]. Another report on MMP-9 involvement in cognition comes from a study on male bipolar patients. Those subjects carrying a *MMP9* gene polymorphism −1562C/C, apparently characterized with lower promoter activity, performed better in cognitive tests than people with other genotypes [132]. It should be, however, noted that recent study by Gregory et al. [133] has questioned the role of this gene polymorphism in regulating MMP-9 expression in the brain and, furthermore, has demonstrated the gene polymorphism affects the brain structure and function that obviously may contribute to cognition.

MMP-3 role in learning and memory is best described in hippocampus-dependent learning tasks. In a spatial memory test—the Morris water maze—it was reported that MMP-3 mRNA and protein levels were elevated during the acquisition phase, while pharmacological inhibition of MMP-3 activity disrupted this learning process [60, 128]. The increase of MMP-3 level in this spatial learning task was prevented by the blocking of the NMDA receptor with the MK-801 antagonist [60, 128]. Similarly to spatial learning, a passive avoidance test revealed an increase of MMP-3 protein level in the hippocampus within a few hours post training, suggesting the role of MMP-3-induced ECM remodeling in associative memory consolidation [134]. Habituation increases MMP-3 expression in hippocampus and PFC [135], while injection of MMP-3 inhibitor into the hippocampus interferes with this task [136].

Looking closer into cellular mechanisms of MMP-3 action in the hippocampus, it was reported that, similarly to MMP-9, a transient activation of MMP-3 (within 30 min window post stimulation) is necessary for synaptic potentiation, described in the excitatory postsynaptic potential (EPSP)-to-spike potentiation phenomenon—a long-term potentiation of synaptic inputs [137]. EPSP-to-spike potentiation is a NMDA receptor-dependent process and the timing of MMP-3 activation overlaps with the short window of NMDARs activity requirement to prolong the potentiation [137, 138]. MMP-3 has been shown to cleave NR1 subunit of NMDA receptors in in vitro neuronal cultures, therefore, it could affect synaptic NMDARs function, necessary during plastic adaptations [139]. Moreover, MMP-3 can affect synaptic efficacy by mediating the structural plasticity of dendritic spines. As reported in the visual cortex of MMP-3 KO mice, the morphology of dendritic spines was impaired, namely, the higher number of short, mature spines perturbed open-eye potentiation in visual cortex of these knockout mice [140].

Similarly to MMP-3, MMP-7 has also been shown to affect spine morphology in a NMDAR-dependent manner. In cultured hippocampal neurons, application of recombinant MMP-7 induced a robust reorganization of spines, changing mushroom-shaped spines into filopodial type. This effect was triggered by rapid F-actin reorganization (observed after 10–20 min) and was dependent on NMDAR function (blocked by MK-801) [122]. In fact, in acute cortical slices, MMP-7 cleaved NR1 and NR2A subunits of NMDARs. This caused a reduction in NMDA-induced Ca^2+^ influx [79]. Therefore, MMP-7 may directly affect NMDARs function and its downstream signaling, pivotal for synaptic plasticity. Moreover, in the presynaptic terminus, application of recombinant MMP-7 on cultured hippocampal neurons decreased the readily releasable pool of synaptic vesicles, decreased the size of active zones and inhibited vesicle recycling [78]. MMP-7 effects on vesicle recycling could be partially explained by its cleavage of the SAP-25 protein (synaptosomal-associated protein of 25 kDa), which disrupts the vesicle docking complex [78].

Among MT-MMPs involved in synaptic functions in the brain, MT5-MMP (MMP-24) is the best characterized. In comparison to other MMPs, it is highly expressed in the brain and it is present at the synapses thanks to binding to AMPA receptor binding protein (ABP), and glutamate receptor interacting protein (GRIP) [141]. Monea and co-workers [141] have also identified N-cadherins as MT5-MMP substrates, which further implies the important function of this MMP at the synapse. Additionally, MT5-MMP is capable of cleaving β-amyloid precursor protein (APP) creating carboxy-terminal fragments of APP, which are able to impair hippocampal LTP as well as reduce neuronal activity in vivo [142]. In a study of traumatic brain injury (TBI) MT5-MMP protein level was increased during synaptic remodeling after the injury and blocking MMPs activity restored levels of N-cadherin and partially restored LTP induction [143]. In those experiments MT5-MMP was, however, expressed mainly in reactive astrocytes and applications of GM 6001, a broad spectrum MMP inhibitor, could not exclude the involvement of other MMPs in this process.

## MMPs in epilepsy and epileptogenesis

Epilepsy is a brain disorder characterized by an enduring predisposition to generate epileptic seizures and by the neurobiological, cognitive, psychological and social consequences of this condition. It is also manifested in the structural changes observed in epileptic brain, such as neuronal reorganization, especially prominent within hippocampus, including axonal sprouting.

In fact, epilepsy is not a homogenous disorder, but rather a collection of subtypes with a variety of etiologies. Thus, epilepsies are divided based on the underlying etiology into: (1) genetic, which are caused by genetic factors; (2) structural/metabolic, which have distinct structural or metabolic conditions, including acquired epilepsies caused by stroke or brain trauma; and, (3) of unknown cause [144, 145]. Acquired epilepsies constitute about 30% of all cases of epilepsy and are most commonly caused by stroke, brain trauma, alcohol use, neurodegenerative diseases, or infection [146]. In acquired epilepsies, brain-damaging insult leads to epileptogenesis (latency period without seizure) lasting up to several years that culminates in the appearance of seizures and epilepsy diagnosis.

Among epilepsy types with not well-defined genetic causes, particularly common are temporal lobe epilepsy (TLE) and post-traumatic epilepsy (PTE). TLE is the most frequent and drug-resistant type of adult focal epilepsy, characterized by the appearance of epileptic foci in such temporal lobe structures as hippocampus or amygdala [147–149]. PTE develops often as a result of TBI caused by an external mechanical force. This initial brain insult triggers a cascade of events called epileptogenesis that can last in humans for years and that finally leads to the appearance of spontaneous recurring seizures (epilepsy) [150].

There are several mechanisms by which MMPs may participate in epileptogenesis and epilepsy, including blood–brain barrier breakdown, and contributions to inflammatory reactions and synaptic plasticity. The best examined proteinase with a significant role in different models and types of epilepsy appears to be MMP-9 [4, 151].

Zhang et al. [152, 153] were the first to report that MMP-9 level (as well as MMP-2) increased in a brain region- and age-related manner in rats subjected to proconvulsive treatment with kainic acid (KA)—a well-recognized model of TLE. Similar increases were also observed after treatment with bicuculline (GABA_A_ receptor antagonist, provoking seizures). Next, Szklarczyk et al. [47] demonstrated that MMP-9 expression—at the levels of mRNA, protein and enzymatic activity—was markedly upregulated by KA. Of particular interest was surprising finding that those responses were limited to the dentate gyrus, i.e., the hippocampal region undergoing the most extensive post-KA plasticity, presumably supporting epileptogenesis. Then, Wilczynski et al. [154] directly investigated the role of MMP-9 in two animal models of epileptogenesis, namely, KA-evoked status epilepticus (SE, a condition known to provoke the development of epilepsy) and proconvulsive pentylenetetrazole (PTZ, a GABA_A_ receptor antagonist) chemical kindling. They showed that the sensitivity to PTZ kindling was decreased in MMP-9 knockout mice but was increased in transgenic rats with neuronal overexpression of MMP-9. Furthermore, Wilczynski et al. [154] demonstrated that MMP-9 deficiency diminished KA-evoked pruning of dendritic spines and decreased aberrant synaptogenesis after mossy fiber sprouting. Finally, they also reported that MMP-9 was associated with excitatory synapses, where both MMP-9 protein levels and enzymatic activity become strongly increased upon seizures. Subsequently, the presumed role of MMP-9 in epileptogenesis was confirmed by Mizoguchi et al. [155], who showed enhanced MMP-9 activity and expression in the injured hippocampus in PTZ kindling model. The role of MMP-9 in the development of epilepsy has been also supported by studies with such models of epilepsy as treatment with either pilocarpine or 4-aminopyridine (seizure-inducing drugs), as well as the use of Wistar Albino Glaxo Rijswijk (WAG/Rij) rats that display higher propensity for seizure activity [156, 157].

PTE is a life-long complication of TBI [158], where seizures are provoked by head injury. Little is known about the mechanisms that lead to post-injury epilepsy development. Based on data from experimental models and human tissue, epilepsy resulting from head injury is associated with various morphological and physiological changes, such as neurodegeneration, myelin and axonal injury, axonal and synaptic plasticity, changes in spine density/morphology, neurogenesis, gliosis, blood–brain-barrier damage, angiogenesis, changes in extracellular matrix and molecular reorganization of ion channels in individual neurons.

It has been reported that in fluid percussion injury (FPI) and controlled cortical impact (CCI) models of TBI, tissue level of mRNA and active form of MMP-9 protein, were increased in ipsilateral cortex and hippocampus after injury [159–162]. This increase was observed during the first-week post-injury [163, 164]. Besides the upregulation of MMP-9, time-dependent increase of its inhibitor, TIMP-1 mRNA has been observed in ipsilateral cortical areas [165]. Unbalance between MMP-9 and TIMP-1 as a result of insult, could induce various structural, cellular and neurochemical changes, such as mossy fiber sprouting, synaptogenesis, changes in expression of β-dystroglycan, BDNF, integrins as well as cell death or neurogenesis. These processes could lead to increased excitability and changes in neuronal circuitry [166]. The possible role of MMP-9 in post-TBI consequences was confirmed by finding that MMP-9 KO mice displayed less prominent motor deficits and significantly smaller post-TBI lesion volumes than wild-type siblings [164]. Recently, Pijet et al. [167] have demonstrated that PTE occurrence is correlated with the size of the lesion upon injury, where the high level of MMP-9 characterized by a large lesion area, predisposes to PTE development, while inhibition of MMP-9, with small lesion volume, protects from PTE. Pijet et al. [167] have also shown that MMP-9 deficiency decreased the seizure appearance in a PTE model, whereas overexpression of MMP-9 increased the likelihood of spontaneous seizures.

The contribution of MMP-9 to epileptogenesis has further been substantiated by the use of MMP-9 inhibitors. It has been shown that inhibitors of MMP-9 used in status epilepticus animal model, reduced neuronal cell death [156, 168, 169], modified the inflammatory response by suppression of pro-inflammatory cytokines in microglial cells [63]. Finally, inhibition of dendritic spines morphological reorganization and severity of seizures induced in kindling model has been observed by Yeghiazaryan et al. [170], who proved the supposition that pharmacological inhibition of MMPs might be beneficial by suppressing seizure progression in animal models of epilepsy [168, 171].

In addition, in humans with different types of epilepsy, the occurrence of the seizures is correlated with the elevated levels of MMP-9 in serum or plasma as well as in the cerebrospinal fluid [172–175]. Similarly, the ratio of MMP-9 to TIMP-1 was increased under epileptic conditions [175]. It has been speculated that this phenomenon can promote brain blood barrier damage [176], which is correlated with elevated expression of MMP-9 in serum [177]. Moreover, studies made on brain surgery tissue revealed increase in MMP-9 level in epileptogenic lesions associated with epileptic conditions such as focal cortical dysplasia (FCD), tuberous sclerosis or TLE [178–182]. Interestingly, in children with intractable and non-intractable epilepsy, salivary MMP-9 concentration was decreased compared to controls [183]. Zybura-Broda et al. [182] have investigated epigenetic changes on the *MMP9* gene promoter, finding an increased DNA demethylation in human epileptic tissue as compared to control brain tissue. Notably, the authors have also demonstrated that progressive *Mmp9* gene promoter demethylation accompanied the development of seizure susceptibility in PTZ kindled rats.

The role of other MMPs in epilepsy development appears poorly investigated. An expression of MMP-2 in epileptogenesis process has often been examined simultaneously with MMP-9. Delayed increase in MMP-2 activity was observed in animal models of TLE after systemic injection of pilocarpine [168] or kainic acid [152, 153]. The increase of MMP-2 was less pronounced compared to MMP-9, nevertheless being significant. However, as so far, there is no further data describing the involvement of MMP-2 in epilepsy development. MMP-2 may increase the permeability of the blood–brain barrier which encourage seizures appearance and facilitates epilepsy development [184]. Interestingly, MMP-2 serum levels in patients with epilepsy were significantly lower [185].

In the kainic acid model of TLE, MMP-3 expression was found to be increased [186, 187]. Moreover, in response to TBI, both hippocampal and CSF levels of MMP-3 were elevated [65, 188]. In contrast, serum MMP-3 levels in epileptic patients was found to be decreased [185]. Elevated expression of hippocampal MMP-3 mRNA and protein after TBI as well as SE was also reported [67, 159, 189]. Gorter et al. [62] also demonstrated an enhancement in the expression of MMP-2 and MMP-14 induced by SE evoked by electrical stimulation of the hippocampus.

Using an unbiased approach of antibody microarrays, Konopka et al. [179] found an elevated expression of MMP-1, -2, -8, -10, and -13, in addition to MMP-9, in the epileptic brain tissue from patients with FCD. However, the expression of these proteinases was not as pronounced and/or not as consistent among patients as the expression of MMP-9. Among these other MMPs, especially striking was the upregulation of MMP-2 in adult patients only, but the significance of this finding is presently unclear.

## MMPs in mental disorders: focus on autism spectrum disorders, fragile X syndrome, schizophrenia, bipolar disorder, chronic stress and major depression

Mental health is as a state of well-being that enables individuals to realize their abilities, deal with the normal stresses of life and work productively [190]. Mental health disorders appear when the homeostasis is lost and abnormalities in thoughts, perceptions, emotions, behavior and relationships with others appear [191]. The rise in number of people suffering from mental disorders makes them a topic of considerable and growing research interest [190]. Importantly, the neuropsychiatric disorders often share comorbidities, hence it may seem difficult to draw a hard line between particular medical conditions [192]. Therefore, it is legitimate to consider that for this reason they also share some aspects of underlying, including molecular, mechanisms. For the purpose of the present review, we shall focus on stress, major depression, bipolar disorder, autism spectrum and schizophrenia– the most common neuropsychiatric disorders. These diseases pose particular research challenges, as they are all difficult to model in experimental animals. In fact, the animal studies usually focus on reproducing and investigating only selective disease symptoms. Fortunately, the very intense effort on human genomics and proteomics has resulted in revealing major categories of possible molecular players underlying the neuropsychiatric conditions. Those categories are often overlapping among those conditions, with the major ones being: inflammatory/immune responses, epigenetics and gene regulation, and finally synaptic plasticity. Considering the fact that MMPs may fall into both inflammatory/immune category and synaptic plasticity and, as it has been well documented for MMP-9 in the brain, may be under very tight, including epigenetic, gene regulation (see above), there is no surprise that MMPs have been implicated, especially MMP-9, by virtue of gene associations and/or protein levels, in neuropsychiatric disorders.

## MMPs in stress and depression

Evolutionarily, stress is a response of the organism to a challenge or threat. When working properly in normal life it helps to maintain balance, and in an emergency situation, to survive. A chronic stress, however, is like a continuous emergency mode that can rewire the brain, so it becomes more vulnerable to anxiety or even a major depression (MD) and other health problems.

### Human data

*MMP9* −1562 C/T (C1562T) gene promoter polymorphism is considered as functional, since a C to T substitution results apparently in a loss of binding of a nuclear silencer protein to this region of the *MMP9* gene to produce an increase in transcriptional activity [193]. This polymorphism has been found to be associated with depression, with the C/C genotype or C allele increasing the risk of susceptibility to middle age depression and the T allele, on the other, hand reducing the risk [194]. The same study also found that in subjects bearing another *MMP9* gene polymorphism of unknown functionality, namely, −1702 T/A, the T/T genotype or T allele led to increase in risk of recurring depressive disorder, whereas the A/A allele led to lower risks. As far as other MMPs are concerned, analysis of *MMP7* −181 A/G gene polymorphism revealed its strong association with higher incidence of recurring depressive disorder. This research also pointed that dual occurrence of C/T C-735T/*MMP2*/genotype and G/G A-181G/*MMP7*/genotype, and, C/T–T/T genotypes of the *MMP2*C-735T and *MMP9*T-1702A polymorphisms, were also associated with increased risks of recurrent depression [194].

Expression analysis of MMPs revealed that MMP-9 was upregulated in serum upon two stressful stimuli (recall of either an event that frustrated the patient or a mental arithmetic task) in patients with coronary disease [195]. In another study, an increased level of MMP-9 was found in the blood of patients with major depression [196]. MMP-9 levels were also correlated with the severity of depression and the quality of life of the patients [197]. MMP-9 levels were also found associated with several psychological factors linked with depression in a middle-aged population [198]. Another interesting observation was that in patients receiving electro convulsive therapy (ECT) as a treatment for depression, the serum levels of MMP-9 decreased significantly in those patients who did not show relapse of depression [199]. Interestingly, transcript and protein levels of MMP-2, MMP-9 and TIMP-2 were found to be downregulated in patients with the recurrent depressive disorder as compared to healthy individuals [130]. Finally, both mRNA and protein levels of several MMPs (MMP2, MMP-7 and MMP-9) were demonstrated to be higher in patients with MD, when compared to healthy controls [200].

### Animal experiments

In an animal model of prolonged stress (restrain of a mouse for 6 h per day, repeated for 21 days) that may contribute to depression, proteolytic activity of MMP-9 was shown to be enhanced in CA1 but not in CA3 of the hippocampus [106]. This result correlated with impaired social interactions that were reinstated following infusion of MMP inhibitor into the hippocampus [106]. Furthermore, Aguayo et al. [201] have found that a single stress exposure (2.5 or 24 h restrain) increases MMP-9 levels and activity in the hippocampus.

## MMPs in autism spectrum disorders and fragile X syndrome

Autism spectrum disorders (ASD) are considered to be neurodevelopmental disorders that affect communication and behavior. They are classified as a “spectrum” as there is varied range of manifestation of the phenotype [202].

### Human data

Probably the most extensively studied genetic alteration linked to ASD concerns *FMR1*, encoding fragile X mental retardation protein (FMRP) [203]. Missing FMRP produces fragile X syndrome (FXS). Most interestingly, MMP-9 was found to be upregulated in the serum of patients with FXS, and, moreover, minocycline treatment that decreased MMP-9 levels led to clinical improvements in the patients [204–207]. In parallel, Janusz et al. [83] have demonstrated that FMRP controls local, dendritic/synaptic translation of MMP-9 and then Gkogkas et al. [208] using *post mortem* brains from FXS patients showed that phosphorylation of the mRNA 5′ cap binding protein, eukaryotic initiation factor 4E (eIF4E), was elevated concomitantly with increased expression of MMP-9 protein. More generally, the analysis of amniotic fluid in patients with ASD showed elevated levels of MMP-9 [209]. Increased MMP-9 was also found in postmortem cortex of ASD patients [210].

### Animal experiments

There are several lines of evidence linking MMP-9 to FXS. This syndrome is well modeled in mice by the knockout (KO) of the FMRP encoding gene (*Fmr1*). In fact, the first link between FXS and MMP-9 was revealed by Bilousova et al. [211], who reported increased MMP-9 activity in cultured neurons from Fmr1 KO mice and, furthermore, the disease phenotype of elongated dendritic spines could be rescued by minocycline inhibiting MMP-9 activity. Similarly, another group also showed that inhibiting MMP-9 could rescue the abnormal spine dynamics noted in Fmr1 KO mice [212]. Most interestingly, genetic removal of MMP-9 rescued the ASD-like symptoms of Fragile X in Fmr1 KO mice [213]. In another study, where abnormally high level of MMP-9 was observed in the auditory cortex of Fmr1 KO mice, genetic deletion of MMP-9 was able to rescue habituation deficits [214].

Furthermore, pharmacological inhibition of elF4E dependent MMP-9 translation (see above) has been able to reverse symptoms of Fragile X in Fmr1 KO mice [208]. In addition, the drug minocycline was able to lower MMP-9 levels in Fmr1 knockout mice, reduced anxiety and rescued Fragile X such as phenotype [215]. Interestingly, metformin, a drug used for treating diabetes, has been shown to normalize elF4E phosphorylation and MMP-9 gene expression, and subsequently rescue the core phenotypes of Fmr1 hemizygous mice [216].

Perineuronal nets (PNN) are assemblies of the extracellular matrix, which cover the interneurons expressing parvalbumin. Abnormalities in PNN have been reported in schizophrenia and Alzheimer’s disease. MMP-9 is considered as a PNN regulator and thus alterations in its levels might result in PNN abnormalities [217]. In fact, genetic reduction of MMP-9 levels to wild-type equivalence in Fmr1 KO mice led to the restoration of PNN formation around parvalbumin cells in the auditory cortex and rescued altered sound-driven response phenotype in these mice [217].

ASD-like-phenotype had also been reported in zinc transporter 3 (ZnT3) KO mice along with increased MMP-9 and BDNF levels—possibly due to perturbed zinc homeostasis. Treatment of these mice with minocycline rescued ASD-like-phenotype and reduced BDNF levels [218].

Notably, it has been suggested that MMP-9 might serve as a very promising target for drugs to treat ASD and FXS patients [219].

## MMPs in schizophrenia and bipolar disorder

Schizophrenia is a neuropsychiatric disorder affecting 0.5% of the human population [220]. The disease is characterized by heterogeneous display of positive symptoms (hallucinations, delusions, and thought disorder), negative symptoms (avolition, restricted affect, poverty of speech, and social withdrawal), and cognitive dysfunction (working memory deficits, executive function, and attentional dysfunction). Schizophrenia onset occurs typically in early adulthood and is usually associated with a lifetime disability. Bipolar disorder (BD) is a major mental disorder with a high risk of suicide [221]. BD onset occurs in adolescence [222]. The disorder is characterized by mania episodes followed by depressive ones [222]. Of note, even though bipolar disorder and schizophrenia are typically considered to be separate diseases entities, in some cases it is difficult to provide a clear separation and these two conditions may be considered as a schizoaffective disorder [223].

### Human data

*MMP9* C1562T gene promoter polymorphism has been shown to associate with schizophrenia [224, 225]. An interesting example of gene-schizophrenia association is rs20544 C/T SNP that has been demonstrated to be strongly linked to the disease delusional symptoms [226]. This polymorphism is located within *MMP9* 3′UTR mRNA and the authors have also shown that it affects RNA structure and binding to FMRP as well as synaptic MMP-9 availability and morphology of dendritic spines [226]. The polymorphism linked to lower MMP-9 synaptic release has been associated with more severe delusional symptoms [226]. Recently, Gregor et al. [227] have shown that the effects of anti-psychotic treatment, as well as the severity of childhood trauma are highly dependent on the type of MMP-9 gene variations. Thus, the SNP rs13925 was found to result in a reduced risk of developing treatment refractory schizophrenia, particularly in the presence of homozygous recessive genotype [227].

As far as *MMP3* is concerned its −1171 5A/6A (rs3025058) polymorphism has been associated with schizophrenia [228]. In this study in Turkish population, it was found that the 6A/6A and 6A allele frequency was less in patients with schizophrenia, whereas the 5A/5A and 5A allele frequency was more.

Measuring the transcript and protein levels of MMPs in schizophrenia has also yielded interesting results. Ali et al. [229] reported that in spite of not finding any correlation between *MMP9* −1562C>T SNP and schizophrenia, the levels of MMP-9 were found to be upregulated in serum of schizophrenic patients. An unbiased approach in screening altered levels of plasma proteins in patients with schizophrenia also revealed increased levels of MMP-9 and TIMP-1 [196]. Rahimi et al. [230] showed that there were no significant differences in the individual levels of MMP-9 and TIMP-1 genes in schizophrenia patients, but the MMP-9/TIMP-1 ratio was significantly altered (see also Ref. [231]). Similarly, upregulated MMP-9 serum levels were found to be strongly associated with levels of mature BDNF (MMP-9 aids in the formation of mature BDNF from pro-BDNF) in schizophrenia patients [232]. Another independent study confirmed increased MMP-9 activity in the blood of schizophrenia patients [233]. Increased MMP-9 serum levels were likewise found to be correlated to oxidative stress in schizophrenia cases [234]. Similarly, MMP-9 gene expression in blood mononuclear cells was found to be upregulated in schizophrenia patients who did not undergo treatment. Interestingly, the levels were no longer significantly different after the administration of the treatment [235].

As in schizophrenia, polymorphisms and changes in the levels of MMPs have been associated with BD. Rybakowski et al. [236] originally demonstrated that C1562T *MMP9* polymorphism linked to BD. It was also found that the serum levels of MMP-9 were upregulated in patients with BD both during the acute phase and remission of depression [237]. Sodersten et al. [238] observed no differences in the level of MMP-9 in patients with BD but found that its levels correlated positively and significantly with mature BDNF levels, what shall be considered in a context of findings suggesting that MMP-9 may cleave pro-BDNF to its mature form.

As far as *MMP10* is concerned, individuals suffering from BD with T/T allele of *MMP10* rs486055 (C/T R53K) polymorphism, were reported to have more depressive events than with C/T or C/C alleles [239]. Interestingly MMP-7 levels were also shown to be elevated in patients with BD [240, 241].

### Animal experiments

Clearly, the cognitive symptoms of schizophrenia appear the easiest to model in animals by studying learning and memory phenomena, which, however, might lead to great oversimplifications. Nevertheless, in this context, it is worthy to recall the aforementioned data linking MMP-9 levels to learning and memory functions (see above). In addition, negative symptoms of the disease are apparently relatively easy to model, however, to dissect those from depressive behaviors might be difficult. The positive symptoms of schizophrenia can be modelled by enhanced hyperlocomotor response to such NMDA receptors antagonists as MK-801. Lepeta et al. [226] demonstrated that MMP-9 heterozygous mice display such enhanced hyperlocomotor response to MK-801, thus, this result was in line with the aforementioned clinical data on MMP-9 3′UTR mRNA polymorphism causing lower synaptic MMP-9 levels and linked to enhanced delusional symptoms of the disease [226].

## MMPs in addiction: alcohol, cocaine, methamphetamine, nicotine

Addiction is a condition characterized by compulsive drug use, seeking and other related behaviors, despite knowledge of the negative consequences [242]. Even though in many cases substance use is the most common type of addiction, a person is capable of developing an addiction also to other rewarding behaviors, such as gambling, game playing or internet as well [243]. Exposure to addictive substances has been shown to create long-lasting alterations in brain function [244]. These take place in brain areas and in neuronal circuits involved in appetitive reward learning and memory formation [245]. The overlap between molecular processes involved in the former and pharmacological actions of drugs suggest that the key proteins driving learning and memory are also important players in the formation of addiction.

The role of MMPs in addiction has recently been reviewed [4, 246]. One of the best described MMPs in the context of addiction is MMP-9. Data from human studies indicate that its levels are increased in the hippocampus of cocaine [247], heroin [248] and alcohol abusers [249]. Furthermore, MMP-9 mRNA level is increased in methamphetamine addicts [250]. Moreover, in alcohol abusers, polymorphism of the MMP-9 gene producing higher protein expression is more frequent in alcoholics’ families than in control subjects’ families [251]. Animal studies revealed the involvement of MMPs in addiction to morphine [252], methamphetamine [76, 253, 254], nicotine [255], ethanol [256–258], cocaine [50, 259, 260] and heroin [50].

MMPs are activated and play functional roles in such particular aspects of addiction, as motivation in mice and human subjects [257], rewarding effect [254, 261, 262] or relapse/reinstatement in rats [50, 255, 259]. Animals devoid of MMP-9 drink as much alcohol as wild-type animals, yet they are impaired in alcohol seeking when access to ethanol is limited (withdrawal) or requires additional effort to obtain it (motivation) [257]. Similar alterations were also observed in MMP-2 and MMP-9 deficient mice, which subjected to methamphetamine treatment did not develop sensitization towards this drug [254].

Some reports indicate that metalloproteinase inhibitors can reduce behavioral correlates of addiction. MMP-2 and MMP-9 inhibitors reduced sensitization and blocked in mice methamphetamine conditioned place preference (CPP), i.e. rewarding effect of drug manifested by time spent in drug-associated chamber [261, 262]. When infused prior to cocaine administration, a broad spectrum MMP inhibitor blocked acquisition of CPP and reduced its reinstatement after a cocaine-priming injection in rats that underwent CPP extinction [259]. The same inhibitor, when injected intracerebroventricularly to rats exposed to long-term alcohol vapor self-administration prevented the escalation of alcohol vapor intake during acute withdrawal [256]. Similarly, in heroin addiction, MMP inhibition attenuates heroin cue-induced seeking [263]. Not surprisingly then, restoring the availability of MMPs reverses these effects, as overexpressing the active form of MMP-9 in the amygdala increased mice motivation towards ethanol self-administration [257].

As far as the structural and functional plasticity is concerned, Smith et al. [50] discovered that extinction and reinstatement of cocaine self-administration causes an increase in spine density, together with the enlargement of spine heads in neurons of the nucleus accumbens, a structure particularly involved in execution of motivated behaviors and addiction [264]. This morphological remodeling is associated with MMP-2 and MMP-9 activities and causes strengthening of glutamatergic synapses, measured by the increase in AMPA/NMDA receptors currents ratio. Our studies in the central amygdala, on the other hand, show that high motivation to ethanol self-administration is associated with the enlargement of long and mushroom spines [257]. This effect is not observed in MMP-9 KO mice. Moreover, ethanol consumption and subsequent withdrawal change synaptic efficacy by the formation of silent synapses and the reduction of AMPA/NMDA receptors currents ratio. Silent synapses are immature synaptic connections, which appearance signifies enhanced plasticity [265]. They are strongly involved in cocaine and morphine addiction [266, 267]. Our observations that MMP-9 activity causes an increase in silent synapse number [29], while genetic ablation of MMP-9 prevents their formation [257], indicate that the function of this gelatinase is particularly significant in the remodeling of synaptic efficacy.

## Concluding remarks

Whereas traditionally, MMPs in the brain used to be considered as enzymes chiefly contributing to a pathological neuronal cell loss occurring in such disorders as various forms of neurodegeneration, stroke, traumatic brain injury, multiple sclerosis, gliomas, etc., the present review offers another look at the brain MMPs. It has been well documented that these enzymes, with MMP-9 serving as a most prominent example (maybe merely because of being the most extensively studied), contribute to learning and memory, as well as such major neuropsychiatric conditions as epilepsy (its development, i.e., epileptogenesis, in particular), schizophrenia, autism spectrum (with particularly strong example of fragile X syndrome), and addiction to various substances of abuse (including alcohol, cocaine, and others), depression, etc. Physiological and pathological synaptic plasticity emerges as a common theme, as far as the mechanisms of all those conditions are concerned. Indeed, the evidence for pivotal role of some MMPs, again MMP-9 in particular, in functional and morphological, physiological and aberrant plasticity of excitatory synapses appears very convincing. The fact that MMP-9 might be locally produced and released at/around those synapses provides further support for this notion. On the other hand, MMPs might be also produced and released by glia and brain-invading leukocytes, fueling a neuroinflammation. To dissect those various activities (including mutual interactions, e.g., during the activation—cleaving off the propeptide—step) of MMPs, their specific expression patterns (cellular origins and time-courses) poses the major research challenge. Addressing this challenge shall advance not only our understanding of the brain in health and disease but may be expected to lead to novel, important disease biomarkers and diagnoses and even new therapies aiming at either inhibiting the enzymes or augmenting their activities. One should not overlook in this context the apparent regulatory and signaling functions of MMPs, their enzymatic nature (particularly amenable for manipulation) and finally, extracellular locus operandi. It has to be finally noted that overlapping substrate specificity of various MMPs poses a great challenge by itself, as far as the development of specific enzyme inhibitors is concerned.

## Abbreviations

3′UTR: 3′ Untranslated region
AMPA: α-Amino-3-hydroxy-5-methyl-4-isoxazolepropionic acid
AMPAR: α-Amino-3-hydroxy-5-methyl-4-isoxazolepropionic acid receptor
APP: β-Amyloid precursor protein
ASD: Autism spectrum disorders
BD: Bipolar disorder
BDNF: Brain derived neurotrophic factor
CA1: Cornu Ammonis area 1, subfield of hippocampus
CA3: Cornu Ammonis area 3, subfield of hippocampus
CAM: Cell adhesion molecule
CCI: Controlled cortical impact
CPP: Conditioned place preference
ECM: Extracellular matrix
ECT: Electro-convulsive therapy
eIF4E: Eukaryotic initiation factor 4E
EPSP: Excitatory postsynaptic potential
FCD: Focal cortical dysplasia
FMRP: Fragile X mental retardation protein
FPI: Fluid percussion injury
FXS: Fragile X syndrome
GABA: γ-Aminobutyric acid
ICAM-5: Intercellular adhesion molecule-5
KA: Kainic acid
KO: Knockout
LTD: Long-term depression
LTP: Long-term potentiation
MD: Major depression
MMP: Matrix metalloproteinase
mRNA: Messenger ribonucleic acid
MT-MMP: Membrane-type MMP
NAc: Nucleus accumbens
NGF: Nerve growth factor
NMDA: *N*-methyl-d-aspartate
NMDAR: *N*-methyl-d-aspartate receptor
PAR1: Protease activated receptor 1
PFC: Prefrontal cortex
PNN: Perineuronal nets
PTE: Post-traumatic epilepsy
PTZ: Pentylenetetrazole
SAP-25: Synaptosomal-associated protein of 25 kDa
SE: Status epilepticus
TBI: Traumatic brain injury
TIMP: Tissue inhibitor of metalloproteinases
TLE: Temporal lobe epilepsy
tPA: Tissue plasminogen activator
uPA: Urokinase plasminogen activator
ZnT3: Zinc transporter 3
